# Poet's Corner

**Published:** 1986-12

**Authors:** Michael Lennard, John Hughes Games, Michael Wilson


### Poet's Corner

**(1) MICHAEL LENNARD 1923–1986**

In his Obituary to Michael Lennard printed in our August number Donald Ratcliffe referred to his love of art and literature. He was a brilliant parodist and we reprint below with kind permission two excerpts from a contribution he made as a student to the 'Black Bag'. The theme was 'hernia' and readers will recognise echoes of Browning's *'Home thoughts from abroad'* and Rupert Brooke's *Grantchester'*. Douglas Tasker, the surgeon referred to in the first poem and a great humorist is remembered with affection by your editor, his one-time Registrar. Incidentally the original was unsigned though the entry on the contents page carried the initials M.B.L. It is possible that some readers now may remember the original 40 years ago and had been unaware of the authorship.

Oh to be in Theatre
Now that Tasker's there;
For he's sure to do a Hernia,
He does hundreds every year.
With a fascial strip from the lateral thigh
He'll repair that hernia by-and-by,
And if you're keen, he'll show you how,
In theatre now.
Show you how and show you well,
For he's the wise surgeon who ties each knot twice over
Lest you should think he never could resuture
That first fine careless rupture.

Just now the Theatre is in use,
And nurses scrub and wash and sluice,
And there in the other room I hope,
Doctors administer their dope,
And patients light and patients deep
Grunt on and on in dreamless sleep.
Oh, there the tables make for you
An ever unforgettable view
Of patients in green gloom who sleep
Ever beneath. The shapeless heap
Surgeons mysterious glide above
In overall white and rubber glove
Ah God, I know it and I know
How the first swabs all golden show
And when the op. is to begin
Gild gloriously the bare skin
That one's to cut . .
God I will fight with might and main
And back to theatre once again,
For that is the only place I know
Where men with useful hands may go,
And of all ops. there I prefer
That nice condition, hernia.

For men with gall stones rarely smile,
Being urban, squat and full of bile,
And as for piles, one cut's enough,
And then ligates and leaves to slough,
And fistulae are mean and dirty,
And no one gets prostates under thirty.
And when they do, they get, I know,
Retention and some overflow,
Necessitating, all over,
At least a daily catheter.
But Hernia, Ah, Hernia!
There's ease of diagnosis there,
For 'cough, please' say, and easily
There is an impulse one can see.
'Herni(a) et virum cano,' and so
Back to the theatre I would go,
For there, ah there, their skins are white
They scrub by day, they scrub by night.
The nurses there do all they should
And only honoraries are rude . .

Ah, God, to see a scalpel thin,
Cut into that golden skin.
To smell the thrilling-sweet and rotten
Unforgettable, unforgotten
Ether smell; to hear the pack
Yell in triumph, 'There's the sac.'

Say, do the dressers crowded stand,
Still watchers white on either hand?
Do bloody swabs in constant flow
Onto their serried hooks still go?
The quiet nurses do they set
All as required of them? Ah, yet
Is there a hernia booked for one
And is there tea when all are done?

**(2) JOHN HUGHES-GAMES**

10th April 1986

'I was sitting disconsolately at the end of an evening surgery recently, having been made aware of a conspicuous gap in the therapeutic armoury. I scribbled the following verses – to be sung to the tune of *There is a green hill far away*'.

*ON THE PASSING OF AN OLD FRIEND*

There is a medicine disallowed,
That I would use galore,
I gave it almost every day,
But now Alas, no more,

There was no other strong enough
To see my patients through
The daily stress and strife of life
As well as it could do.

So if a patient had bad nerves
Or if they had the flu,
'Twas really wonderful to see
What a dose or two could do.

O sadly, sadly is it missed
And sad to see it gone,
That universal toner-up
*MIST, POT, BROM, ET NUX VOM.*

**(3) MORE PRESIDENTIAL CLERIHEWS**

1983–1984

Dr Norman Tricks
Has his practice in the Styx,
And when it gets too hot
He lives upon his yacht.

1984–1985

Dr Ian Bailey
Does his exercises daily,
And you can see the reason
When he gets his skis on.

1985–1986

Dr Jose Jancar
Must occasionally hanker
For a patient with a brain
On an intellectual plane.

M. G. Wilson.

1986–1987

Mr Huw Griffith
Through our pages giveth
To a problem his solution
With the Imtran Revolution. (see p 29)

*NOTE – Clerihews on the three past Presidents – Dr Norman Brown, Dr Robert Warin and Mr Herbert Bourns were printed in 1983. I was surprised, though perhaps I should not have been, to find how many people had never heard of this 'art form'. I was stimulated to attempt them by the publication of 'The 'first Clerihews' by E. Clerihew Bentley. (Oxford University Press, 1982) They were defined as Biography for beginners, and they were compiled into a 'Dictionary of Biography' in 1893. The first, and possibly best known, 'came to him' at the age of 16.*

*Sir Humphrey Davy*
*Abominated gravy.*
*He lived in the odium*
*Of having discovered sodium.*

*There were 60 or 70 of them and they were reproduced in their original form in Bentley's handwriting with illustrations by G. K. Chesterton who also, with several other named cronies, wrote some of them. Lesser known and among my favourites are*

*John Milton*
*Preferred Cheddar to Stilton,*
*He placed the Devil*
*On an entirely new level.*

*It was not Napoleon*
*Who designed the Ashmolean.*
*He never had the chance,*
*Living mostly in France.*

